# Reduction of the Spatial Stroop Effect by Peripheral Cueing as a Function of the Presence/Absence of Placeholders

**DOI:** 10.1371/journal.pone.0069456

**Published:** 2013-07-24

**Authors:** Chunming Luo, Juan Lupiáñez, María Jesús Funes, Xiaolan Fu

**Affiliations:** 1 Key Laboratory of Behavioral Science, Institute of Psychology, Chinese Academy of Sciences, Beijing, China; 2 Mind, Brain and Behavior Research Center, University of Granada, Granada, Spain; University of Bath, United Kingdom

## Abstract

In a paradigm combining spatial Stroop with spatial cueing, the current study investigated the role of the presence vs. absence of placeholders on the reduction of the spatial Stroop effect by peripheral cueing. At a short cue-target interval, the modulation of peripheral cueing over the spatial Stroop effect was observed independently of the presence/absence of placeholders. At the long cue-target interval, however, this modulation over the spatial Stroop effect only occurred in the placeholders-present condition. These findings show that placeholders are modulators but not mediators of the reduction of the spatial Stroop effect by peripheral cueing, which further favor the cue-target integration account.

## Introduction

The spatial coding of location is an important cognitive skill. Consequently, how its mental representation is built up and how this representation affects organization of actions have attracted considerable research interest. Spatial coding of this type has been examined in a variety of conflicting tasks used to examine spatial congruency effects, including stimulus-stimulus (S-S) congruency effects, such as the spatial Stroop effect (SSE), and the stimulus-response (S–R) congruency effect, as in the case of the Simon effect [1]–[3].

In a version of the spatial Stroop task, an up or down-pointing arrow appears randomly above or below a fixation point. Although participants are asked to discriminate the direction of the arrow while ignoring its location, they typically make faster and more accurate responses to congruent stimuli (i.e., an up-pointing arrow located above the fixation sign) than to incongruent ones (i.e., a down-pointing arrow located above the fixation sign) [4], [5].

In a Simon task, responses are usually faster and more accurate when the stimulus appears in the same relative location as the response, even if the stimulus location is irrelevant to the task [3], [6]. These spatial congruency effects suggest that the spatial location coding of an object is automatic, as it usually affects performance even when the location is entirely irrelevant to the defined task [5], [7].

Results from previous studies have indicated that the occurrence of spatial pre-cues indicating one of the possible target locations has an effect on spatial congruency effects, which have been interpreted as modulation of peripheral cueing on the building up of spatial code representations, with weaker spatial codes at attended than unattended locations [2], [8]. Based on this finding, some studies therefore have further investigated whether spatial cueing can modulate the magnitude of spatial congruency effects such as the Simon effect [8]–[10] or the spatial Stroop effect [4], [5], [7], [11]–[13]. One of the most robust findings has been the systematic reduction in S-S spatial congruency effects on peripherally cued as compared to peripherally uncued location trials. By contrary, S–R spatial congruency effects seem to be unaffected by peripheral spatial cueing.

Several accounts have been proposed to explain this modulation of spatial attention over spatial congruency effects. According to the attention shift account [6], [14]–[16], the reduction of spatial congruency effects on cued trials might be due to the fact that attention shifts create spatial codes relative to the prior position of attention. Therefore, if attention has been moved towards the cued location preceding the target appearance, no attentional shift toward the target location would be necessary when the target is presented. Therefore, no spatial code would be created for the target, and consequently a null Simon or spatial Stroop effect should be observed at attended locations.

According to a revised version of the referential coding account, Danziger and colleagues [7] argued that, within the context of the combination of spatial cueing paradigm and the spatial Stroop task, the spatial location of target may be coded left–right relative to two simultaneous objects of reference: the central fixation point object and the lateralized spatial cue object. On oppositely cued trials, the target location would be coded relative to both the left–right cued location and the central location, while on cued trials, the target location would be left–right coded only relative to the central point, because it would be coded as “same” relative to the spatial cue. Consequently, this explanation would also predict a reduction of spatial congruency effects for cued trials compared to oppositely cued trials.

Although these two explanations could account for the pattern of spatial Stroop reduction on cued trials, they can not explain why such an effect is absent for S–R congruency effects such as Simon or compatibility effects [5], [17]. Also, these two explanations can not explain other related results such as the finding that the spatial Stroop effect is only reduced with the presence of peripheral cues, while it is increased when the arrow location is cued by centrally presented cues that endogenously orient attention to the indicated location [4]. Finally, the reduction of spatial Stroop has been observed on cued trials compared to no-cue trials [11].

Considering the whole set of data regarding the modulation of spatial cueing on the spatial Stroop effect [4], [5], [11], [17], Funes et al. [4] and Lupiáñez and Funes [5] claimed that this modulation could be better explained by an alternative explanation, i.e., the object file integration account proposed by Lupiáñez and colleagues for discussions of event integration processes in exogenous cueing contexts [18], [19]. According to this account, an abrupt onset (peripheral cue) can operate as a perceptual object or event [20], [21] when it is contiguous in time with the target and shares the common spatial location with it. Assuming that spatial and temporal contiguity play an important role in event or object integration processes [22]–[24], the facilitation effect often observed at short cue–target stimulus onset asynchronies (SOAs) could be attributed, at least in part, to rapid integration of the spatial codes for the cue and the target when they occur close together in both time and space [11], [25]–[27].

These authors assume that the integration of cue and target spatial codes within the same event or object file means that no extra spatial code is created when the target appears, as it will not be treated by the perceptual system as a new object, for which a new location code would be needed, but as an update of the object representation triggered by the cue, to which only the direction information is added. This integration process thus helps to separate in time the processing of the two conflicting dimensions (the spatial location and its direction) of the target stimulus, as the distracting location dimension of the arrow target links with an event (the cue) that occurred at an earlier point in time. The separation in time of these two perceptual codes could then underlie the reduction in the spatial congruency effect observed for valid trials, as the irrelevant location dimension would have largely decayed by the time the relevant direction dimension was coded (see [28], for discussion of a similar temporal overlap hypothesis as it applies to Simon interference). Note that the cue–target event integration would not occur when the cue and target appear at different locations, as would be the case for uncued location trials following peripheral noninformative cues and for no-cue trials [11].

The strongest evidence favoring this view comes from two recent studies [17], [29] that directly investigated the separate role of object-based and space-based selection on S–S and S–R congruency effects by combining the double-rectangles cueing paradigm developed by Egly, Driver and Rafal (1994) [30] with the arrow version of the spatial Stroop task. The authors presented a display with two vertically or horizontally arranged objects (rectangles), one of which was cued at one side, and left/right pointing arrow targets were presented in one of four critical conditions: either at the cued location (same location), at an uncued location in the cued object (same object), at an uncued location in the uncued object (different object), or at the location diametrically opposite the cued end (far location). With the objects placed as far apart as they were long, there were equal distances between the cued and the uncued locations for the same vs. different object conditions. Results showed that mean RTs were faster in same location condition than in the other conditions, indicating that location or distance from the cue affected performance (i.e., a space-based effect was observed). Furthermore, responses were faster for same object targets than for different object targets, notwithstanding their equivalent distance from the cued location, indicating that the rectangle object also had an influence on the allocation of attention (i.e., an object-based effect was also observed). Importantly, the spatial Stroop or S-S congruency effects (but not the S-R congruency) was reduced in the same-object condition, as compared to the different-object condition, while no further reduction was observed at the same-location condition, indicating that the occurrence of cue and target in the same rectangle reduced the spatial Stroop effect, which was attributed to the contribution of the rectangle to the cue-target integration process in the same-location or same-object conditions.

The fact that the rectangle object had an influence on the allocation of attention and its modulation over the spatial Stroop effect [17], [29], might render it unclear whether cue-target integration processes are directly responsible for the reduction of spatial Stroop by peripheral cueing or this integration need to be mediated by the rectangle object. It is important to note that placeholders were used in all the above reviewed literature concerning the role of attention on the spatial Stroop effect [4], [5], [7], [11], [12], [18], [19], [25].

**Figure 1:**
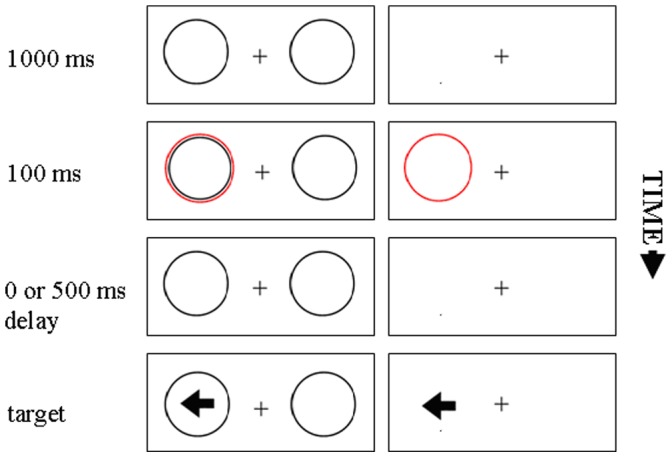
The basic trial sequence used in Experiment 1. The left column represents placeholder present condition and the right column represents placeholder absent condition.

The main purpose of the present study was to examine whether the spatial Stroop effect would be reduced by peripheral cueing in the absence of placeholders, and whether this reduction varies with the SOA between the cue and the target (see the right column in Figure 1). If peripheral cueing reduces the spatial Stroop effect in this situation, we would add confirming evidence that cue-target integration is directly responsible for the reduction of spatial Stroop by cueing, and this integration need not to be mediated by placeholders.

Moreover, we compared a situation where placeholders were absent, to a condition where placeholders were present along the whole trial (see Figure 1). At a short SOA between cue and target we expected to replicate the previously observed reduction of the spatial Stroop effect at the cued location, even when no placeholders are present, given that at the short SOA the location codes of the cue and the target would be easily integrated within the same object-file, because they occurred closely together in time. Importantly, however, at a longer SOA, the reduction of spatial Stroop effect by cueing might only occur in the placeholder-present display, because the cue and target appearing in the same placeholder helps the integration of cue and target within the same object file, manifesting an object-based effect, as observed in Luo et al. [17], [29]. These results together would show that the placeholders are a modulator of cue-target integration extending in time the window for integration.

For the current manipulation, the attentional shift account will predict the spatial Stroop effect to be smaller on cued than uncued trials for both the placeholder present and absent conditions, because a similar shift of attention towards the cue should occur at the short and long cue-target intervals, provided that the cueing effect is facilitatory in both conditions, as observed in the previous studies with placeholders present [4]–[5].

Also, Danziger et al. (2001)’ referential coding account will predict the spatial Stroop effect to be smaller on cued than uncued trials for both the placeholder present and absent conditions and invariant of SOAs, given that target is coded left–right relative to the central fixation cross and the cue on cued trials, but is coded left-right relative to the central fixation cross on uncued trials.

## Experiment 1

The basic method of the experiment is straightforward, placeholder boxes were present on half of trials but they were absent on the other half. Thus, cues and targets could appear either in objects or in an essentially blank display. We mainly wanted to investigate whether cueing would modulate the spatial Stroop effect in the placeholder-absent condition, and whether it would do in a similar or different way as it does in the placeholder-present condition reviewed above.

### Method

#### Participants

Eighteen undergraduate students (7 males and 11 females) were paid to participate in this experiment. All participants had normal or corrected-to-normal vision and were naïve as to the purpose of the experiment. Written consent was obtained from all participants prior to participation. The protocol was approved by the institutional review board (IRB) at the institute of psychology, Chinese Academy of Sciences.

#### Apparatus and procedure

Stimuli presented on a super VGA high-resolution color monitor. A computer, running E-Prime 1.1 software, controlled the presentation of stimuli, timing operations, and data collection. Participants viewed the monitor from a distance of 57 cm in a dimly lit room.

For all trials, the display sequence in a trial differed depending on whether placeholder boxes were present. Two typical trial sequences for each display type are illustrated in Figure 1. The background of the display was white and all stimuli were black except for the red cue. In addition, the empty circular cue (3.6°× 3.6°, stroke, 0.1°), empty circular boxes (each 3.2°×3.2°, stroke, 0.1°), and target arrow (1.7°×1.5°) were presented in the middle of the screen and were centered 3.8° from the center of the screen, respectively.

Each trial began with a central fixation cross (0.8°×0.8°) and two circular boxes separately located at the left and right of it for placeholder-present display but only with the cross for placeholder-absent display. After 1 s, a red cue flickered at the left or righ of the fixation cross with equal probability for 100 ms. Following a further interval of 0 or 500 ms from the cue offset (depending on the SOA), the imperative arrow appeared, which was left or right pointing. The target remained visible until the subject responded or for 1000 ms if no response was emitted. Then the next trial began. The interval between trials was 500 ms and the screen remained white throughout this interval.

#### Design

There were two sessions of 544 trials each, with a rest interval of 5 min between them. Each session consisted of two large blocks corresponding respectively with placeholder-present and placeholder-absent conditions with a rest interval of 60 s between them and their order was randomized. Each block included one practice block of 16 trials followed by two test blocks of 128 trials. Each test block corresponded with one SOA and their order was random.

All participants were instructed to complete the two sessions of trials. Responses were made with the index fingers of both hands, pressing the C and M keys on the computer keyboard for left and right responses, respectively. In one session of trials, the response location was compatible with the direction of arrow, i.e., pressing the C key when the arrow was left pointing and pressing the M key when it pointed right, regardless of the arrow’s location, while the reverse mapping was used in the other session of trials, on which the response location was incompatible with the direction of the arrow, i.e., pressing the M key when the arrow was left pointing and pressing the C key when it pointed right, regardless of the arrow’s location. The order of the two sessions was counterbalanced across participants. The response keys and computer screen were aligned such that the fixation point and the midway point between the two response keys were on the participant’s sagittal midline. Participants were firmly instructed to maintain fixation and to respond to the targets as quickly and accurately as possible.

The experiment had a 2 (display type: placeholder-present, placeholder-absent)×2 (cueing: cued, cued)×2 (spatial Stroop: congruent, incongruent)×2 (compatibility: compatible, incompatible)×2 (SOA: 100 ms, 600 ms) design, with 32 observations per experimental condition. Compatibility refers to whether the location of arrow and response location are compatible, and spatial Stroop refers to whether the location of arrow and its direction are congruent.

### Results

#### RT analysis

Mean reaction times (RTs, in ms) and percentage errors (PEs) are presented in Table 1. The ANOVA on RTs revealed main effects of three variables, cueing, *F*(1, 17) = 50.53, *p*<.001, spatial Stroop, *F*(1, 17) = 18.17, *p = *.001, and compatibility, *F*(1, 17) = 6.12, *p = *012. Cueing interacted with spatial Stroop, *F*(1, 17) = 38.22, *p*<.001, and SOA, *F*(1, 17) = 6.82, *p* = .018. The interaction between spatial Stroop and compatibility also was significant, *F*(1, 17) = 7.35, *p* = .015. There were three-way interactions of cueing, spatial Stroop, and SOA, *F*(1, 17) = 5.89, *p* = .027, display type, cueing, and spatial Stroop, *F*(1, 17) = 9.29, *p* = .007, and display type, spatial Stroop, and SOA, *F*(1, 17) = 11.63, *p* = .003. No other effects were significant. To disentangle these interactions, and in order to more closely test our hypotheses, we performed a separate ANOVA for each SOA, with display type, cueing, and spatial Stroop, and compatibility as within-subjects variables.

**Table 1:** Experiment 1: Mean Reaction Time (in ms) and percentage errors (in Parentheses) as a function of display type, cueing, SOA, spatial Stroop and compatibility.

	Placeholder Present				Placeholder Absent			
	100 ms SOA		600 ms SOA		100 ms SOA		600 ms SOA	
	Cued	Uncued	Cued	Uncued	Cued	Uncued	Cued	Uncued
CC	416(1.6)	420(1.9)	427(1.6)	430(2.1)	422(2.1)	433(2.8)	421(2.3)	428(3.5)
CI	442(2.3)	446(1.4)	446(1.4)	453(3.3)	444(1.9)	452(2.3)	446(2.3)	450(1.4)
IC	434(3.0)	463(5.0)	437(2.8)	460(5.7)	433(4.3)	461(5.4)	440(4.2)	451(3.6)
II	437(2.1)	478(4.5)	443(2.6)	465(3.6)	443(2.3)	475(3.0)	457(4.0)	471(3.6)

CC* = *Congruent & Compatible, CI = Congruent & Incompatible, IC = Incongruent & Compatible, II = Incongruent & Incompatible.

**Figure 2:**
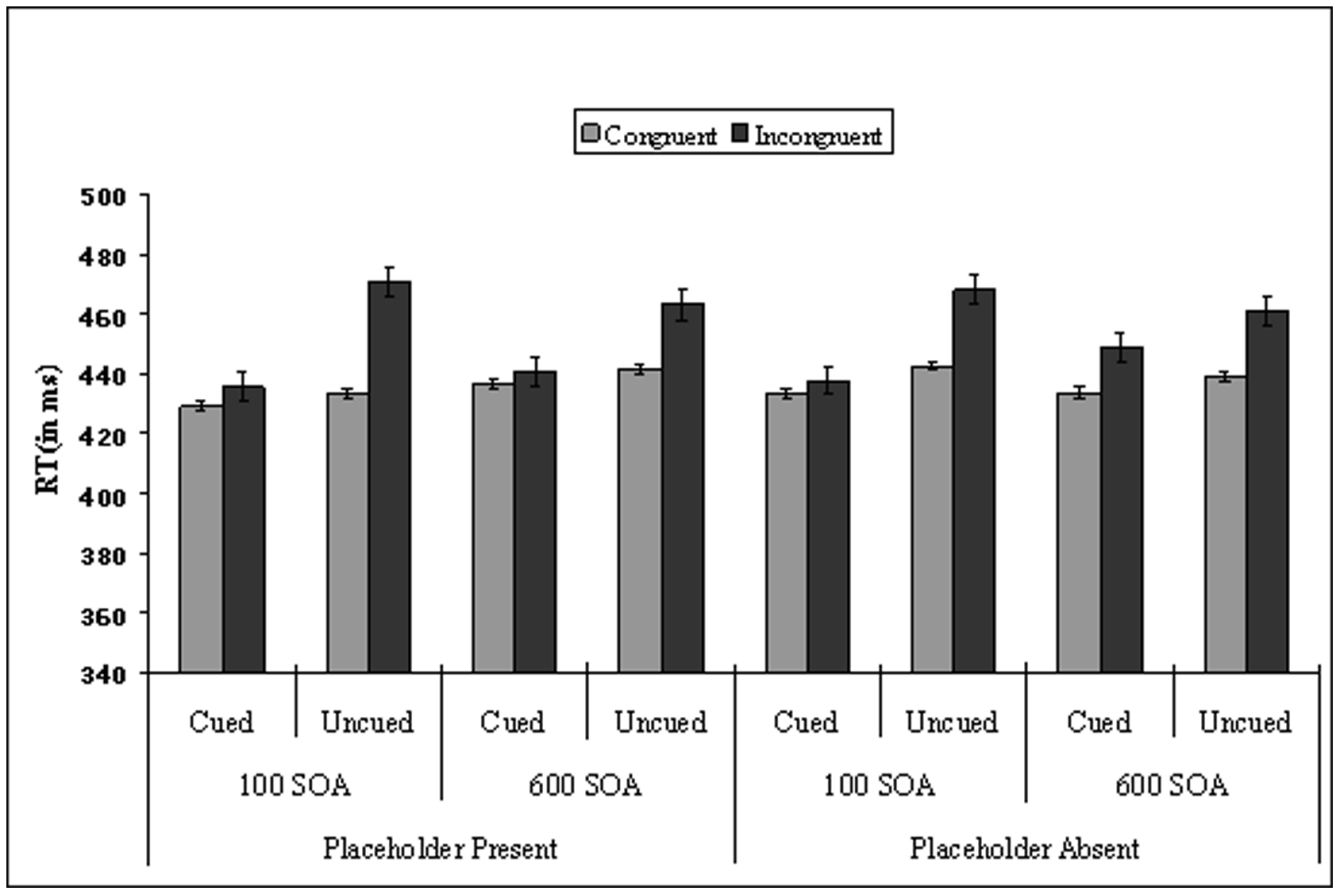
Mean Reaction Time (in ms) as a function of display type, cueing, SOA, and spatial Stroop in Experiment 1.

Errors. sented in Table 1. ed than uncued trials. ant, tibility, and the three-way interaction was not significant. The analysis performed on the short SOA data revealed that cueing, *F*(1, 17) = 66.06, *p*<.001, spatial Stroop, *F*(1, 17) = 24.36, *p*<.001, and compatibility, *F*(1, 17) = 6.07, *p* = .025, were significant. Spatial Stroop interacted with compatibility, *F*(1, 17) = 4.49, *p* = .049. Importantly, as shown in Figure 2, cueing interacted with spatial Stroop, *F*(1, 17) = 33.50, *p*<.001, with a smaller spatial Stroop effect (6 ms) on cued than uncued location trials (32 ms), while the three-way interaction between display type, Cueing and spatial Stroop was not significant, *F*(1, 17) = 2.93, *p* = .105. However, the interaction between cueing and compatibility was not significant (*F*<1), and neither were the interaction between display type, cueing and compatibility and the only four-way interaction (*Fs*<1). No other effect was significant.

The analysis for long SOA revealed that cueing, *F*(1, 17) = 13.61, *p* = .002, and spatial Stroop, *F*(1, 17) = 11.05, *p* = .004, were significant. The main effect of compatibility was marginally significant, *F*(1, 17) = 4.41, *p* = .051, and it interacted with spatial Stroop, *F*(1, 17) = 8.04, *p* = .011. Importantly, as shown in Figure 2, cueing interacted with spatial Stroop, *F*(1, 17) = 8.05, *p* = .011, and the interaction between display type, cueing, and spatial Stroop was marginally significant, *F*(1, 17) = 3.48, *p* = .080. In order to more clearly test our predictions, a separate ANOVA for each display type was then performed, with cueing, spatial Stroop, and compatibility as within-subjects variables. The analyses of the placeholder-absent data showed that cueing, *F*(1, 17) = 5.26, *p* = .035, and spatial Stroop, *F*(1, 17) = 10.83, *p* = .004, were significant. As shown in Figure 2, cueing did not interact with spatial Stroop (*F*<1). In addition, the two-way interactions cueing and compatibility, and spatial Stroop and compatibility were not significant (*F*s<1), and neither was their three-way interaction, *F*(1, 17) = 1.28, *p* = .275. No other effect was significant.

The analysis of the placeholder-present data showed that cueing, *F*(1, 17) = 20.94, *p*<.001, and spatial Stroop, *F*(1, 17) = 8.92, *p* = .008, were significant. As shown in Figure 2, cueing interacted with spatial Stroop, *F*(1, 17) = 15.74, *p* = .001, with a smaller spatial Stroop effect (4 ms) on cued than uncued location trials (21 ms). However, the two-way interaction between cueing and compatibility was not significant, and neither was the three-way interaction between cueing, spatial Stroop and compatibility (*F*s<1). No other effect was significant.

#### PE analysis

In the error analysis, there was a main effect of cueing, *F*(1, 17) = 4.52, *p = *.048, and of spatial Stroop, *F*(1, 17) = 19.07, *p<*.001. No other effects were significant.

### Discussion

As in the previous studies [4]–[5], facilitation effects were observed for the placeholder-present display even at the 600 ms SOA. In this experiment, we found that the same was true for the placeholder- absent display, as the cueing effects did not revert into IOR at the long SOA. Moreover, for the placeholder-present display, the modulation of the spatial Stroop effect by cueing occurred not only at the short but also at the long SOA, while for the placeholder-absent display, this modulation just occurred at the short SOA, even though cueing effect was facilitatory at the both SOAs. Thus, placeholders are not necessary for the modulation of cueing over the spatial Stroop effect to be observed, although they seem to modulate this effect, perhaps by extending in time the window for integration.

Also replicating previous studies [4]–[5], for the placeholder-present display, the compatibility effect was not modulated by cueing, regardless of SOA, as was the case with the placeholder-absent display. These findings, together with the modulation of cueing over spatial Stroop, confirm again that the modulation of cueing over spatial congruency effects takes place at perceptual- related stages of processing, but not at response-related stages of processing.

## Experiment 2

In experiment 2, we used a vertical display (i.e, up/down pointing arrows appearing above/below fixation) to see whether the pattern of results observed in experiment 1 would occur in a procedure where only spatial Stroop (i.e., S-S congruency) is measured. This change allowed to measure a pure spatial Stroop effect that results from the conflict of the direction of arrow and its location without the confound of the conflict between arrow location and response location, given that the responding hand (whether left or right) was orthogonal to the location and direction of the arrow (top/bottom, up/down) [4]–[5], [31].

### Method

#### Participants

Twenty-four undergraduate students (10 males and 14 females) were paid to participate in this experiment. All participants had normal or corrected-to-normal vision and were navïe as to the purpose of the experiment. Written consent was obtained from all participants prior to participation. The protocol was approved by the institutional review board (IRB) at the institute of psychology, Chinese Academy of Sciences.

#### Apparatus, procedure and design

The apparatus were identical to the experiment 1. The procedure was identical to the experiment 1, apart from the following changes: Participants only completed one session. The target was an arrow pointing either up or down, and the display was vertical. For half participants, the task was to press the C key (left response) when the arrow pointed up, and the M key (right response) when it pointed down, regardless of the arrow’s location above or below fixation, while the reverse mapping was used for the other half participants.

The experiment had a 2 (display type: placeholder-present, placeholder-absent)×2 (cueing: cued, cued)×2 (spatial Stroop: congruent, incongruent)×2 (SOA: 100 ms, 600 ms) design, with 32 observations per experimental condition.

### Results

#### RT analysis

Mean RTs and PEs are presented in Table 2. The ANOVA on RTs revealed main effects of two variables, cueing, *F*(1, 23) = 25.47, *p*<.001, and spatial Stroop, *F*(1, 23) = 57.01, *p*<.001. Cueing interacted with spatial Stroop, *F*(1, 23) = 29.26, *p*<.001. Moreover, the interaction between cueing, spatial Stroop, and SOA was significant, *F*(1, 23) = 5.59, *p* = .027, and the display type×cueing×spatial Stroop three-way interaction was marginally significant, *F*(1, 23) = 3.99, *p* = .058. No other effects were significant. As in Experiment 1, to disentangle these interactions and more closely test our hypotheses, we performed a separate ANOVA for each SOA, with display type, cueing, and spatial Stroop as within-subjects variables.

**Table 2:** Experiment 2: Mean Reaction Time (in ms) and percentage errors (in Parentheses) as a function of display type, cueing, SOA, and spatial Stroop.

	Placeholder Present				Placeholder Absent			
	100 ms SOA		600 ms SOA		100 ms SOA		600 ms SOA	
	Cued	Uncued	Cued	Uncued	Cued	Uncued	Cued	Uncued
Congruent	471(3.6)	469(2.9)	469(2.6)	474(5.1)	478(4.0)	483(5.2)	474(5.5)	487(4.4)
Incongruent	487(4.7)	513(5.6)	483(5.3)	508(8.1)	492(6.2)	522(7.4)	503(6.5)	514(8.5)

**Figure 3:**
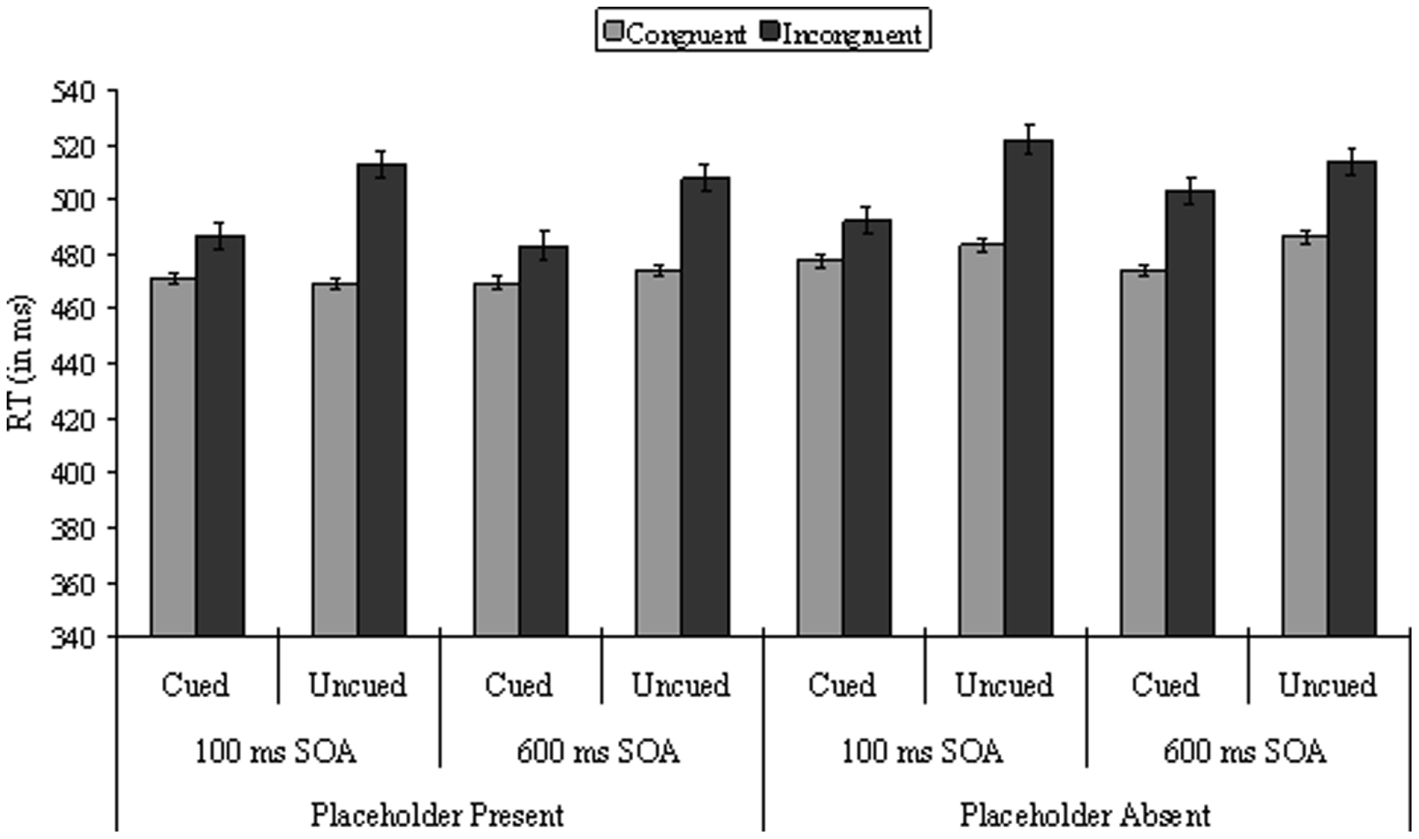
Mean Reaction Time (in ms) as a function of display type, cueing, SOA, and spatial Stroop in Experiment 2.

The analysis showed main effects of two variables, cueing, *F*(1, 23) = 21.85, *p*<.001, and spatial Stroop, *F*(1, 23) = 67.20, *p*<.001. As shown in Figure 3, the interaction between cueing and spatial Stroop was significant, *F*(1, 23) = 26.76, *p*<.001, with a smaller spatial Stroop effect (15 ms) on cued than uncued location (42 ms), but the three-way interaction between display type, cueing and spatial Stroop was not significant (*F*<1). No other effects were significant.

The analysis showed again main effects of two variables, cueing, *F*(1, 23) = 14.15, *p* = .001, and spatial Stroop, *F*(1, 23) = 32.14, *p*<.001. The interaction between cueing and spatial Stroop was not significant, *F*(1, 23) = 2.55, *p* = .124, but the three-way interaction between display type, cueing, and spatial Stroop was significant, *F*(1, 23) = 5.94, *p* = .023. A separate ANOVA for each display type was then performed, with cueing and spatial Stroop as within-subjects variables. The analysis of the placeholder-absent data showed that the main effects of cueing, *F*(1, 23) = 6.68, *p* = .017, and spatial Stroop, *F*(1, 23) = 31.54, *p*<.001, were both significant. However, as shown in Figure 3, cueing did not interact with spatial Stroop (*F*<1; the spatial Stroop effect was 29 and 27 ms respectively for cued and uncued location trials). In contrast, the analyses of the placeholder-present data showed that cueing, *F*(1, 23) = 10.54, *p*<.001, and spatial Stroop, *F*(1, 23) = 22.00, *p*<.001, were also significant, but, importantly, as shown in Figure 3, their interaction was significant, *F*(1, 23) = 7.01, *p* = .014, with a smaller spatial Stroop effect (14 ms) on cued than uncued location (34 ms).

#### PE analysis

The ANOVA on PEs revealed a main effect of cueing, *F*(1, 23) = 14.13, *p = *.001, and of spatial Stroop, *F*(1, 23) = 24.68, *p*<.001. Cueing interacted with spatial Stroop, *F*(1, 23) = 9.75, *p* = .005, with a smaller spatial Stroop effect (1.8%) on cued than on uncued location trials (3.7%). No other effects were significant.

### Discussion

As in Experiment 1 and the previous studies [4]–[5], a facilitation effect was observed for the placeholder-present display even at the 600 ms SOA. The same was true for the placeholder-absent display, as the cueing effects did not revert into IOR at the long SOA. Moreover, for the placeholder-present display, the reduction of the spatial Stroop effect by cueing occurred at both the short and long SOA, while for the placeholder-absent display, this modulation only occurred at the short SOA, even though the cueing effect was also significant at the long SOA.

## General Discussion

The current study investigated the role of the presence/absence of placeholders on the reduction of the spatial Stroop effect by peripheral cueing. We found that the typical spatial Stroop modulation by cueing occurred in both the placeholder present and absent conditions at the 100 ms SOA between the cue and the target. Such modulation survived up to 600 ms SOA when placeholders were present, while no modulation was observed at this SOA when placeholders were absent.

As reviewed in the introduction, there exist three possible hypotheses that have been used to explain the reduction of spatial Stroop by peripheral cueing. According to the *attention shift account* [14]–[15], the spatial code is generated contingent on the last shift of attention. Therefore, once attention has been moved towards the cued location, no spatial codes are created for targets appearing at this location, and therefore no conflict should arise in the same location condition between the irrelevant target location and its relevant direction. This account can accommodate the findings of a smaller spatial Stroop effect at the cued than at the uncued location at both the short and the long SOA for the placeholder-present display, and at the short SOA for the placeholder-absent display. However, this account would predict no effect of the presence/absence of placeholders as a function of SOA, and therefore it would have problems explaining the differential effect of cueing over spatial Stroop observed at the longer SOA as a function of the presence/absence of placeholders, with no modulation being observed in the absence of placeholders. It has also problems explaining previous findings that attentional shift within the same object does not modulate the spatial Stroop effect [17], [29].

Similarly, the *referential coding* account [7] could be compatible with the findings of a smaller spatial Stroop effect at cued than uncued location at the short SOA, as the target would be coded relative to the fixation cross when the target appeared at the same location as the cue, but relative to both the fixation cross and the cue when it appeared at the opposite uncued location. However, the *referential coding* account might have more problems to explain why this modulation depends on the interaction between the presence/absence of placeholders and SOA. Similarly this account can not explain other results described in the introduction as the fact that spatial Stroop is also reduced on cued location trials compared to a neutral condition where no extra visual cue is presented [11].

All these findings, however, thoroughly reconcile with the event integration account [4], [5]. According to this account, with valid cues, due to the spatial and temporal contiguity between the cue object and the arrow target, they could integrate together within the same object [24]. It has been recently shown that location (i.e., spatial overlap) is enough for the integration of features into the same object file [32]. Therefore, integration occurring on valid trials leads to the target not being coded as appearing either left or right of fixation in experiment 1, or either above or below it in experiment 2. Instead, it would be coded in both cases as appearing within the object file opened by the cue, which would be updated including the direction information provided by the arrow target. Therefore, no new object representation is created when the target appear, and therefore no location code is activated simultaneously with processing of direction information, which results in the reduction of the spatial Stroop effect observed on validly cued trials. For invalid trials, however, a new object file must be created when the target appears, thus leading to simultaneous activation of location and direction codes and therefore regular spatial Stroop effects.

Interestingly, the event integration account also predicts that no IOR would be observed in a difficult discrimination task, as the one used in the current experiments. According to this account, IOR is considered as a cost in detecting new information at a location where a peripheral cue appeared before (i.e., at a location where an object representation was just opened) [26]–[27]. However, the fact that no new object representation is to be created when the target arrow appear, as the already opened object representation is updated with the arrow direction information, is measured as a benefit in the discrimination of the direction of the target arrow. However, it would be measured as a cost if the target were to be detected; in contrast to uncued location trials, on cued location trials there would be no signal (i.e., no opening of new object representation) marking the onset of a new object to be detected.

Altogether, we may conclude that the reduction of spatial Stroop by peripheral cueing might arise from cue-target integration processes. More importantly, we report evidence showing that the temporal window for object-file integration can be extended in time. The significant reduction of spatial Stroop observed at the 600 ms SOA when placeholders are presented (but not when they are absent) provides evidence that cue-target integration, although reduced, can survive up to 600 ms. Evidence for a similar extension of the temporal window for integration was observed by Akyürek, Toffanin, and Hommel (2008) in an Attentional Blink paradigm. In this case the temporal extension, rather than being stimulus-driven as in our case, depended on the expectation about the duration of the first stimulus to be integrated. The expectation for a longer duration of the first stimulus (T1) increased its integration with the following stimulus (T2). In a different study by Chica, Charras, and Lupiáñez (2008) [33] it was shown that temporal integration in a cue target procedure also depended on the set participants had to either detect or discriminate the target. They used the Illusory Line Motion procedure (a peripheral cue is integrated with a target line producing the illusion of the line moving away from the cue) to show that the illusion could be reduced (i.e, the temporal window for integration could be reduced) when participants adopted an attentional set to detect the line, in comparison to a set to discriminate it.

Therefore, these results together with the evidence reported in the current paper support the role of spatio-temporal overlap in object-file integration processes. Whereas integration occurs quite automatically as a direct consequence of this spatio-temporal overlap, the temporal window for integration can be modulated both endogenously [34] and exogenously, as shown in the current paper. Future research should investigate the interaction between endogenous and exogenous factors in modulating the temporal window for integration. To the extent that stimuli (i.e., placeholders in our case) are presented bridging the gap between the stimuli opening the object-file representation (the cue in our case) and the stimuli adding more information to the object (arrow direction in our case) we predict an important role of endogenous factors. Perhaps the role of endogenous factors is much more moderate when no stimuli are presented to bridge the gap.
